# Coronavirus Disease 2019-Induced Tracheomegaly: A Case Report

**DOI:** 10.7759/cureus.23810

**Published:** 2022-04-04

**Authors:** Saiara Choudhury, Asad Chohan, Pahnwat T Taweesedt, Rahul Dadhwal, Abhay Vakil

**Affiliations:** 1 Internal Medicine, Corpus Christi Medical Center, Corpus Christi, USA; 2 Department of Pulmonology, Corpus Christi Medical Center Bay Area, Corpus Christi, USA; 3 Pulmonary Medicine, Corpus Christi Medical Center, Corpus Christi, USA; 4 Internal Medicine, University of North Texas, Denton, USA

**Keywords:** computed tomography (ct) imaging, tracheobronchomegaly, tracheomegaly, chronic cough, covid 19

## Abstract

Tracheomegaly is a medical condition where the tracheal diameter is greater than the upper limits of normal. Tracheomegaly can be classified as primary or secondary. Primary tracheomegaly is usually congenital. Secondary tracheomegaly can be due to multiple causes, including connective tissue disease, infections, autoimmune diseases like sarcoidosis, and prolonged mechanical ventilation. Here, we describe the first reported case of tracheomegaly secondary to coronavirus disease 2019 (COVID-19) pneumonia and COVID-induced interstitial lung disease (ILD). While many cases of tracheomegaly are asymptomatic, patients can have symptoms like cough, dyspnea, hemoptysis, or even respiratory failure. Tracheomegaly is associated with a higher risk of recurrent lower respiratory tract infections, chronic cough, bronchiectasis, and tracheobronchomalacia. Early recognition of COVID-19-induced tracheomegaly can help initial early management and reduce the incidence of infections.

## Introduction

Tracheomegaly is a medical condition where the tracheal diameter is greater than the upper limits of normal. For coronal and sagittal diameters, these values are defined in men aged between 20 and 79 as 25 mm and 27 mm and in women as 21 mm and 23 mm, respectively [1]. Tracheomegaly can be classified as primary or secondary. Primary tracheomegaly is usually congenital. Secondary tracheomegaly can be due to multiple causes, including connective tissue disease, infections, autoimmune diseases like sarcoidosis, and prolonged mechanical ventilation [2,3].

Here, we describe a case of tracheomegaly secondary to coronavirus disease 2019 (COVID-19) pneumonia and COVID-induced interstitial lung disease (ILD).

## Case presentation

**Figure 1:**
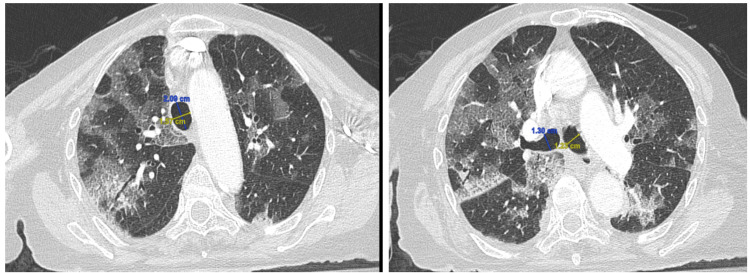
Computed tomography (CT) of the chest highlighting the sagittal (blue) and coronal (yellow) diameters of the trachea (left) and at the level of tracheal bifurcation (right) highlighting the diameter of the right and left bronchi

**Figure 2:**
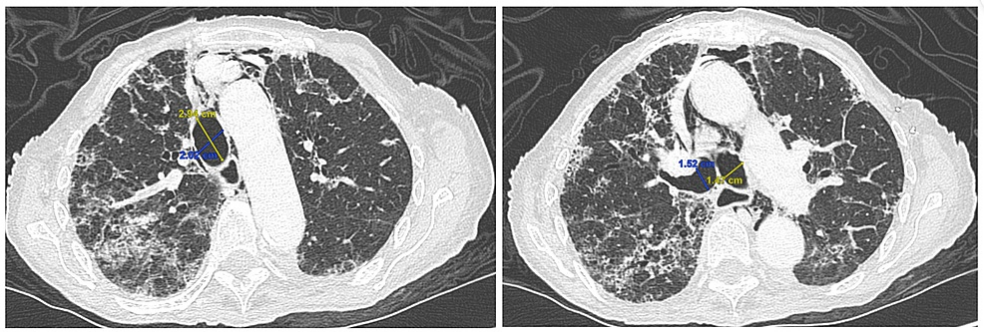
Computed tomography (CT) of the chest obtained six months after COVID-19 infection highlighting the increase in sagittal (yellow) and coronal (blue) diameters of the trachea (left) and at the level of tracheal bifurcation (right) highlighting the diameter of the right and left bronchi COVID-19: coronavirus disease 2019

An 89-year-old female presented to the emergency department with worsening shortness of breath associated with pleuritic chest pain for one week. The patient reported that she was diagnosed with COVID 19 pneumonia six months ago and was admitted to the hospital with shortness of breath and hypoxia. Her hospital course was complicated with mild pneumomediastinum secondary to COVID 19 pneumonia. She was discharged home with supplemental oxygen without the need for non-invasive or invasive mechanical ventilation. During these six months, she required 2 L of oxygen via nasal cannula and reported a persistent cough with occasional production of green-yellow sputum. Her physical exam revealed a frail female who appeared stated age and was not in any acute distress. Respiratory exam showed rales in bilateral lung bases. Laboratory studies were unremarkable. Computed tomography (CT) of the chest showed pneumomediastinum, parenchymal changes consistent with fibrosis, traction bronchiectasis (Figure 2) when compared to CT of the chest six months prior (Figure 1) when the patient was admitted to the hospital with COVID 19 pneumonia. The images also show apparent enlargement of the trachea from a sagittal diameter of 20 mm to 29 mm in six months, and an enlargement of the sagittal diameter of the left main bronchi from 12 mm to 14 mm and the right main bronchi from 13 mm to 15 mm. The sagittal cuts of CT images six months prior during the first encounter and the images obtained during the second encounter are shown in Figures 1-2, respectively.

The patient was prescribed chest physiotherapy, bronchodilator therapy, and mucolytic agents to improve pulmonary hygiene and induce clearance of respiratory secretions. After a discussion with the patient and her family, she was discharged home with hospice care per the patient’s wishes.

## Discussion

Airway abnormalities can cause symptoms such as cough, shortness of breath, or wheezing [3]. Tracheobronchomegaly (TBM) describes the phenomenon of marked dilation of the trachea and primary bronchi [4]. Specifically, it is defined as transverse and sagittal tracheal diameters exceeding 25 and 27 mm, respectively, or the left and right mainstem bronchi exceeding 18 or 21 mm in diameter, respectively, in men. In women, the values are 21 and 23 and 17.4 and 19.8 mm, respectively [1,3]. While it is more commonly seen in the pediatric population, many cases have been described in the adult population. Primary TBM is congenital and described in diseases such as Mounier-Kuhn, Ehlers-Danlos, and Marfan syndromes [3,4]. Secondary TBM has various etiologies and has been described in multiple case studies. Parris and Johnson described a case of TBM secondary to radiotherapy for the treatment of oropharyngeal carcinoma [5]. A study by Woodring et al. in 1989 analyzed 34 chest radiographs of patients with diffuse pulmonary fibrosis. Ten (29%) patients were found to have TBM. Out of these patients, seven were found to have progressive TBM on subsequent imaging [6]. Multiple case reports have described TBM as secondary to prolonged invasive mechanical ventilation [7]. A recent case report described a case of TBM related to tracheostomy in a patient with respiratory failure due to COVID-19 [2].

Many radiographic and CT findings have been described in patients with post-COVID syndrome, including but not limited to ground-glass opacities and reticular opacities with or without parenchymal distortion, which could suggest developing fibrosis [8]. In our patient, the CT images from acute COVID-19 infection to six months later show many changes described in the literature regarding post-COVID syndrome, including ground-glass opacities, interstitial thickening, reticular parenchymal changes suggesting fibrosis. However, the images also show the development of TBM in the span of six months. Previous literature has not reported these CT findings related to COVID-19 infection. In our patient, the tracheal diameter enlargement meets the criteria for tracheomegaly. However, while present, the degree of bronchial enlargement does not meet numerical criteria for the definition of bronchomegaly. However, given prior studies showing TBM progression in patients with fibrosis, follow-up CT imaging may play a role in monitoring TBM progression in patients with post-COVID-19 lung disease [6].

TBM results from atrophy of elastic fibers and thinning of muscle of the tracheobronchial tree. The enlarged tracheobronchial tree leads to an inefficient cough mechanism that impedes mucociliary clearance and leads to mucous retention that can cause bronchiectasis, recurrent pneumonia, and eventually, pulmonary fibrosis [9]. Many patients with TBM may remain asymptomatic. Symptomatic patients often have cough, dyspnea, hemoptysis, and even respiratory failure [9]. Given the CT findings in our patient, it is crucial to understand the clinical implications of secondary TBM. A retrospective study by Schmitt et al. studied 17 patients diagnosed with TBM due to Mounier-Kuhn syndrome and found that the most common associated respiratory conditions included recurrent infections and bronchiectasis. The most common non-respiratory comorbid condition among these patients was the presence of gastroesophageal reflux disease [10]. Similarly, in the study by Woodring et al., all the patients with TBM secondary to pulmonary fibrosis had a higher incidence of chronic cough and repeated lower respiratory tract infections [6].

In the era of the COVID-19 pandemic where more than 100 million people have been infected with the severe acute respiratory syndrome coronavirus 2 (SARS CoV-2), these findings are significant because the eventual development of TBM as part of COVID-19 sequelae can lead to an increased risk of recurrent pulmonary infections in patients who have had COVID-19 infection [11]. Additionally, TBM can also have unexpected implications during preprocedural general anesthesia. A case report by Lee et al. describes a patient undergoing abdominal surgery who was orally intubated during the procedure and found to have a significant peritubal air leak that could not be eliminated despite reinflation and using a larger size endotracheal tube. The patient was found to have incidental tracheomegaly and tracheobronchomalacia with hyperdynamic collapse and copious secretions on flexible bronchoscopy. The tracheomegaly was thought to be the reason for the persistent air leak. The surgery had to be postponed and was later done using regional instead of general anesthesia [9]. Thus, given all these possible TBM complications, monitoring patients who have been infected with COVID-19 infection for whom TBM should be considered.

The management of TBM includes a combination of bronchodilators, inhaled corticosteroids, chest physiotherapy, pulmonary rehabilitation, and long-term oxygen therapy [10]. Tracheal stenting and tracheobronchoplasty are also treatment options in patients with symptomatic TBM [12].

## Conclusions

TBM or tracheomegaly is a rare phenomenon that has primary and secondary causes. Our case report highlights a patient with chronic cough and shortness of breath with a history of COVID-19 infection, who developed symptomatic tracheomegaly secondary to COVID-19 infection. Given the clinical implications of TBM, including the risk of recurrent lower respiratory tract infections, development of chronic cough, and increased complications with anesthesia, it is essential to monitor patients who have been infected with COVID-19 for development or progression of TBM.

## References

[1] Breatnach E, Abbott GC, Fraser RG (1984). Dimensions of the normal human trachea. Am J Roentgenol.

[2] Harper S, Robinson M, Manning G, Jones A, Hobson J, Shelton CL (2020). Management of tracheostomy-related tracheomegaly in a patient with COVID-19 pneumonitis. Anaesth Rep.

[3] Marom EM, Goodman PC, McAdams HP (2001). Diffuse abnormalities of the trachea and main bronchi. Am J Roentgenol.

[4] Dutau H, Cavailles A, Fernandez-Navamuel I, Breen DP (2009). Tracheal compression in a patient with Marfan's syndrome-associated tracheomegaly treated by an XXL stent: the largest diameter airway stent ever placed in a previously undescribed airway condition. Respiration.

[5] Parris WC, Johnson AC (1982). Tracheomegaly. Anesthesiology.

[6] Woodring JH, Barrett PA, Rehm SR, Nurenberg P (1989). Acquired tracheomegaly in adults as a complication of diffuse pulmonary fibrosis. Am J Roentgenol.

[7] Kucuk C, Arda K, Ata N, Turkkani MH, Yildiz ÖÖ (2016). Tracheomegaly and tracheosephagial fistula following mechanical ventilation: a case report and review of the literature. Respir Med Case Rep.

[8] Solomon JJ, Heyman B, Ko JP, Condos R, Lynch DA (2021). CT of post-acute lung complications of COVID-19. Radiology.

[9] Lee CC, Lin BS, Chen JY, Chuang CC (2017). Anesthesia for a patient with unexpected giant tracheobronchomegaly. Tzu Chi Med J.

[10] Schmitt P, Dalar L, Jouneau S (2016). Respiratory conditions associated with tracheobronchomegaly (Mounier-Kuhn Syndrome): a study of seventeen cases. Respiration.

[11] (2022). CDC COVID Data Tracker. https://covid.cdc.gov/covid-data-tracker/.

[12] Krustins E, Kravale Z, Buls A (2013). Mounier-Kuhn syndrome or congenital tracheobronchomegaly: a literature review. Respir Med.

